# Supplementary material for: Carbon metabolic rates and GHG emissions in different wetland types of the Ebro Delta
Source: PLoS One. 2020 Apr 22;15(4):e0231713. doi: 10.1371/journal.pone.0231713 (PMC7176097; doi:10.1371/journal.pone.0231713)
Supplement: S1 Table — (DOCX) [file pone.0231713.s001.docx]

|  |  | **Depth**  **(cm)** | **Oxygen**  **(mg L^-1^)** | **Oxygen Sat.**  **(%)** | **T**  **(ºC)** | **Cond.**  **(mS cm^-1^)** | **pH** | **Chl-*a***  **(mg m^-3^)** | **TSS**  **(mg L^-1^)** | **Alk (meq L^-1^)** | **SRP**  **(mmol m^-3^)** | **NO_3_^-^**  **(mmol m^-3^)** | **NH_4_^+^**  **(mmol m^-3^)** |
| --- | --- | --- | --- | --- | --- | --- | --- | --- | --- | --- | --- | --- | --- |
| ALFA | Average | **44.5** | **6.9** | **87.0** | **22.2** | **56.6** | **8.1** | **5.0** | **70.3** | **4.0** | **0.2** | **33.7** | **13.3** |
|  | Standard deviation | 38.4 | 2.4 | 51.2 | 8.5 | 16.7 | 0.3 | 2.5 | 20.5 | 1.8 | 0.3 | 6.1 | 4.7 |
|  | Max | 100.0 | 10.6 | 125.0 | 32.9 | 78.8 | 8.6 | 9.7 | 101.3 | 9.1 | 0.9 | 41.5 | 20.3 |
|  | min | 15.0 | 1.9 | 28.7 | 8.1 | 30.3 | 7.7 | 2.0 | 37.8 | 3.0 | 0.0 | 22.3 | 7.6 |
| ENCA | Average | **19.5** | **7.1** | **69.0** | **20.5** | **31.3** | **8.0** | **22.5** | **61.4** | **4.2** | **0.2** | **26.6** | **19.4** |
|  | Standard deviation | 1.6 | 3.2 | 22.7 | 9.6 | 21.2 | 0.2 | 20.8 | 40.5 | 2.0 | 0.3 | 8.1 | 12.2 |
|  | Max | 20.0 | 10.9 | 87.7 | 29.2 | 64.5 | 8.4 | 60.3 | 137.9 | 9.9 | 1.0 | 37.8 | 39.5 |
|  | min | 15.0 | 2.9 | 38.4 | 4.3 | 5.4 | 7.7 | 2.7 | 8.5 | 3.1 | 0.0 | 13.7 | 9.4 |
| FBIO | Average | **53.0** | **7.1** | **68.7** | **20.0** | **2.0** | **7.9** | **19.6** | **29.9** | **4.2** | **0.1** | **8.5** | **7.8** |
|  | Standard deviation | 2.6 | 2.6 | 15.5 | 8.9 | 0.5 | 0.3 | 8.8 | 11.3 | 1.0 | 0.1 | 3.8 | 6.5 |
|  | Max | 55.0 | 11.6 | 91.4 | 28.7 | 2.7 | 8.2 | 36.3 | 43.5 | 6.7 | 0.3 | 13.0 | 16.7 |
|  | min | 50.0 | 4.4 | 56.5 | 5.4 | 1.1 | 7.4 | 9.3 | 14.7 | 3.4 | 0.0 | 0.0 | 1.9 |
