# Supplementary material for: Carbon metabolic rates and GHG emissions in different wetland types of the Ebro Delta
Source: PLoS One. 2020 Apr 22;15(4):e0231713. doi: 10.1371/journal.pone.0231713 (PMC7176097; doi:10.1371/journal.pone.0231713)
Supplement: S2 Table — (DOCX) [file pone.0231713.s002.docx]

|  |  | **Cond.**  **(mS cm^-1^)** | **pH** | **Eh** | **Water content**  **(%)** | **LOI**  **(%)** | **Carbonate**  **(%)** |
| --- | --- | --- | --- | --- | --- | --- | --- |
| ALFA | Average | **45.8** | **8.0** | **-51,3** | **64.4** | **34.2** | **17.2** |
|  | Standard deviation | 15.4 | 0.3 | 119,3 | 9.0 | 25.0 | 5.9 |
|  | Max | 71.9 | 8.5 | 141,1 | 79.9 | 65.5 | 25.3 |
|  | min | 32.8 | 7.6 | -175,2 | 51.6 | 13.1 | 9.9 |
| ENCA | Average | **36.2** | **7.6** | **-131,2** | **67.0** | **28.1** | **17.6** |
|  | Standard deviation | 16.8 | 0.3 | 74,3 | 9.3 | 10.9 | 2.8 |
|  | Max | 67.0 | 8.1 | -16,1 | 79.8 | 38.4 | 21.1 |
|  | min | 17.4 | 7.2 | -206,2 | 58.8 | 15.6 | 14.7 |
| FBIO | Average | **1.7** | **7.6** | **-63,2** | **23.1** | **3.9** | **15.7** |
|  | Standard deviation | 0.7 | 0.4 | 43,2 | 3.3 | 0.7 | 0.5 |
|  | Max | 3.1 | 8.2 | 15,6 | 28.2 | 4.6 | 16.3 |
|  | min | 0.8 | 7.1 | -111,0 | 17.8 | 2.6 | 14.6 |
