# Supplementary figures and images for: Carbon metabolic rates and GHG emissions in different wetland types of the Ebro Delta
Source: PLoS One. 2020 Apr 22;15(4):e0231713. doi: 10.1371/journal.pone.0231713 (PMC7176097; doi:10.1371/journal.pone.0231713)

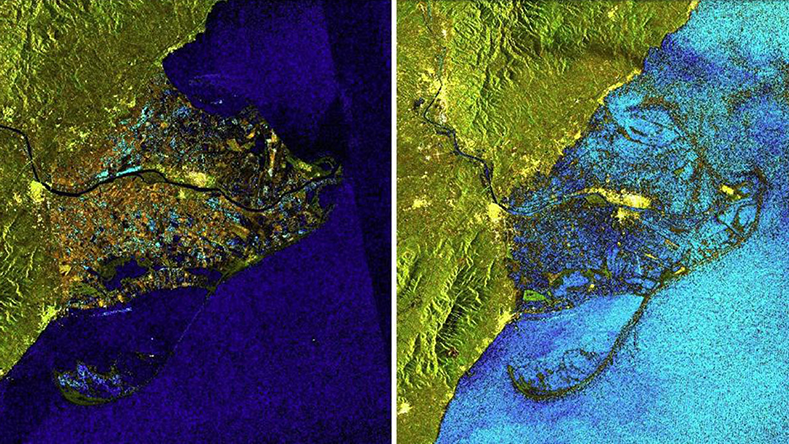

Supplement: S1 Fig — Source: Copernicus Services. (TIF) [file pone.0231713.s003.tif]
